# Supplementary material for: Barriers to access and unmet needs in mental health care for Venezuelan migrants in a southern border region of Colombia: The experiences of community workers
Source: PLOS Ment Health. 2026 Apr 10;3(4):e0000597. doi: 10.1371/journal.pmen.0000597 (PMC13068237; doi:10.1371/journal.pmen.0000597)
Supplement: S1 File — (DOCX) [file pmen.0000597.s001.docx]

Interview topic guide

The following are suggested questions to use as a framework, but the aim is to use them flexibly and to follow as far as possible the interviewee’s priorities.

Tell me about your job and the type of work you do with Venezuelan migrants.

Do you work primarily with migrants in transit or with those seeking to remain?

What types of mental health problems do you see among the migrants you work with? How do you identify these problems?

Please tell me about the services that currently exist for migrants with mental health problems in this region.

What works well and helps you connect people with services?

Please tell me about the barriers you have faced in your work when trying to connect migrants with health services to get help for mental health problems.

Tell me how you feel about these barriers? How does this work affect you?

Is there any major unmet need that you encounter in your work related to the mental health of the migrant population?

Prompts to consider:

Differences between transit and settled populations

Most vulnerable groups

Migration status

Health service affiliation

Knowledge of their rights

Stigma, discrimination

Substance use

Migrants’ attitudes toward seeking medical help
