# Supplementary material for: Barriers to access and unmet needs in mental health care for Venezuelan migrants in a southern border region of Colombia: The experiences of community workers
Source: PLOS Ment Health. 2026 Apr 10;3(4):e0000597. doi: 10.1371/journal.pmen.0000597 (PMC13068237; doi:10.1371/journal.pmen.0000597)
Supplement: Attachment — Submitted filename: Responsetoreviewers.PLOSMH.18Feb2026.docx [file pmen.0000597.s003.docx]

Division of Population Health, Health Services Research and Primary Care

School of Health Sciences

Faculty of Biology, Medicine and Health

The University of Manchester

United Kingdom

18 February 2026

Dear Reviewers,

Re: PLOS Mental Health submission, revisions following review.

**Barriers to access and unmet needs in mental health care for Venezuelan migrants in a southern border region of Colombia: the experiences of community workers.**

Thank you for taking the time to review our manuscript. We are grateful for your detailed and insightful comments which we believe have helped us to strengthen the manuscript.

For clarity, we respond to each comment in turn.

Reviewer 1

1. **Clarify and strengthen the conceptualization of what is defined as “access to mental health care”.** We have added in lines 97-102 the definitions we are using of access to care, barriers and unmet needs.

2. **Concerns regarding the overgeneralization of psychoactive substance use.** Thank you for alerting us to what we agree is a risk that our findings could be misinterpreted. We have added a paragraph (lines 517-524) which states the need for caution in interpreting the findings, which should not be used to feed discriminatory stereotypes, and stresses the need for further research.
3. **Acknowledge and contextualise the imbalance in participant representation with predominance of humanitarian-sector participants.** This is addressed in the discussion of theme four (lines 551-555) and in Limitations (lines 579-591).
4. **Minor stylistic and grammatical edits are recommended.** We have attempted to improve the manuscript in this manner. This is visible in the version with tracked changes.
5. **Concerns over the use of “ignorance” to describe provider-level barriers.** We agree that this term may imply individual failings, which was not our intention. Therefore, “ignorance” has been changed to phrases such as limited awareness, knowledge gaps and lack of knowledge, depending on the context (seen in tracked changes).

Reviewer 2

1. **The need for a clearer definition and stronger justification of “community workers”.** Our definition of community workers in this study – as a general term, including a range of frontline activities rather than a single, specific profession – is explained in lines 129-141. Further details about the work of the participants recruited (remembering the need to preserve anonymity in a small local workforce) is provided in lines 249-256. The strength of choosing this population is explained in lines 121-128. Our decision not to interview migrants directly (for practical and ethical reasons) is explained in lines 142-147.
2. **Align the methodological claim of “phenomenology” with what is studied.** We agree that our original manuscript had a tension between the declared phenomenological approach and the fact that the results do contain some second-hand accounts of the experiences of migrants. This was the result of what the participants were keen to share during the interviews. We feel that our overall approach is phenomenological, and that a strength of our study is the focus on the lived experiences of the community workers in facing their challenges. However, we believe that second-hand accounts can still be relevant when the focus is on their impact on the community workers. We have explained this in lines 152-159. We have also made adjustments in the results section to emphasise how second-hand accounts are being used.
3. **Strengthen inclusion and exclusion criteria.** We have added further information in lines 134-141 and 166-178. We have tried to present this in a logical way without unnecessary repetition. Specifically, we explain that we only excluded healthcare clinicians (doctors, nurses, psychologists) in order to explore non-specialist views of mental health. We did not exclude frontline workers who had received training in mental health, who, as you mention, are a common and important part of the workforce.
4. **Explain why community workers are the primary participants.** This is addressed in answer 1 above.
5. **Clarify novelty.** We agree that what were themes 3 and 4 (substance misuse, reduction of services) are more original and potentially stronger. We have restructured the article to reflect this, moving these themes to positions 1 and 2 (and adjusting the abstract and discussion accordingly). We have made some developments to these themes in the discussion (visible in tracked changes). We have also slightly shortened the other themes (now 3 and 4), accepting that they are more confirmatory but arguing that they still contribute insights and nuance to established evidence.
6. **Interview guide** has been added as supplementary material.
7. **Reflexive Thematic Analysis.** Thank you for pointing out the methodological incongruities in our approach, in particular the employment of practices which appear post-positivist rather than in keeping with RTA. The Braun and Clarke 2025 reference you provided on RTARG was very helpful, and we have used this alongside Braun and Clarke’s 2024 paper “Supporting best practice in reflexive thematic analysis reporting in Palliative Medicine: A review of published research and introduction to the Reflexive Thematic Analysis Reporting Guidelines (RTARG)” (both papers now cited in the manuscript). We feel that RTA is our overriding approach in this study, and the best approach for the research question and data, so we have revised the methodology (in particular on participant selection 188-192, data analysis 206-222, reflexivity 241-246), results and discussion sections to reflect this. In particular, we have removed “validity checks” such as triangulation, while still contextualising our findings in established evidence in a way supported by RTARG.

We thank you again for your attention to our article and look forward to receiving your further comments.

Yours faithfully

John Fitton (University of Manchester; currently University College London)

Julie Benavides-Melo (Universidad Cooperativa de Colombia campus Pasto)

Christine Greenhalgh (University of Manchester)
