# Supplementary material for: Barriers to access and unmet needs in mental health care for Venezuelan migrants in a southern border region of Colombia: The experiences of community workers
Source: PLOS Ment Health. 2026 Apr 10;3(4):e0000597. doi: 10.1371/journal.pmen.0000597 (PMC13068237; doi:10.1371/journal.pmen.0000597)
Supplement: Attachment — Submitted filename: Responsetoreviewers.PLOSMH.19Mar2026.docx [file pmen.0000597.s004.docx]

Division of Population Health, Health Services Research and Primary Care

School of Health Sciences

Faculty of Biology, Medicine and Health

The University of Manchester

United Kingdom

19 March 2026

Dear Editor and Reviewers,

Re: PLOS Mental Health submission, minor revision following review.

**Barriers to access and unmet needs in mental health care for Venezuelan migrants in a southern border region of Colombia: the experiences of community workers.**

Thank you for your further review of our manuscript.

In response to your comment in the correspondence of 12^th^ March 2026, we have added a conceptual definition of “access to healthcare” in lines 97-102. The reference for this definition has been added, and the numbers of subsequent references have been adjusted accordingly. This is a commonly cited definition of healthcare access and is well aligned with our study because it combines both service and population factors.

Thank you for your attention and please do let us know if any further revisions are required prior to acceptance.

Yours faithfully

John Fitton (University of Manchester; currently University College London)

Julie Benavides-Melo (Universidad Cooperativa de Colombia campus Pasto)

Christine Greenhalgh (University of Manchester)
